# Controls on coastal flooding in the southern Baltic Sea revealed from the late Holocene sedimentary records

**DOI:** 10.1038/s41598-022-13860-4

**Published:** 2022-06-11

**Authors:** Karolina Leszczyńska, Karl Stattegger, Damian Moskalewicz, Robert Jagodziński, Mikołaj Kokociński, Przemysław Niedzielski, Witold Szczuciński

**Affiliations:** 1 Geohazards Research Unit, Institute of Geology, Adam Mickiewicz University, Bogumiła Krygowskiego 12, 61-680 Poznań, Poland; 2 Department of Geomorphology and Quaternary Geology, University of Gdańsk, Bażyńskiego 4, 80-952 Gdańsk, Poland; 3 Hydrobiology Department, Faculty of Biology, Adam Mickiewicz University, Uniwersytetu Poznańskiego 6, 61-614 Poznań, Poland; 4 Faculty of Chemistry, Adam Mickiewicz University, Uniwersytetu Poznańskiego 8, 61-614 Poznań, Poland

**Keywords:** Natural hazards, Environmental impact

## Abstract

Climate change and related sea-level rise pose significant threats to lowland coasts. However, the role of key controlling factors responsible for the frequency and landward extent of extreme storm surges is not yet fully understood. Here, we present a high-resolution sedimentary record of extreme storm surge flooding from the non-tidal southern Baltic Sea, spanning two periods: 3.6–2.9 ka BP and 0.7 ka BP until present. Sediments from coastal wetland, including sandy event layers, were analyzed by sedimentological (grain size, loss-on-ignition, micromorphology), geochronological (^14^C), geochemical (XRF), mineralogical (heavy minerals) and micropaleontological (diatoms) methods. The results show that both periods were characterized by high-frequency of storm surge flooding, in order of 1.3–4.2 events per century. These periods correlate with phases of enhanced storminess in northwest Europe and took place during both rising and fluctuating sea levels. The study shows that the frequency and landward extent of coastal inundation, largely depended on the development of natural barriers (e.g. beach ridges and aeolian foredunes). Thus, in the context of the future coastal storm-surge hazard, the protection of existing coastal barriers and their morphology is essential.

## Introduction

The stability of the global coastline, in particular of lowland coasts is threatened by climate change and rising sea level along with regionally enhanced frequency and intensity of storms^1–4^. Consequently, lowland coasts face a period of instability and more frequent storm flooding. Projections of future condition of coastal areas range from those forecasting catastrophic loss of sandy beaches with rising sea level to those projecting relatively modest adjustment of coastal morphology and ecosystems under moderate rates of sea-level rise^5^. However, commonly accepted is that unstable climatic conditions and sea-level change result in rising uncertainty in predicting the occurrence of coastal flooding^3,6^.

Extreme storm water level causing storm surge flooding is associated with a rapid rise in the sea level due to the influence of wind, waves and atmospheric pressure (including seiche effect) on the sea surface, combined with tides. The storm surges on the coastline may be classified into four regimes: swash (wave runup remaining below the elevation of the dune foot), collision (the runup reaches the dune foot), overwash (beach berm and/or dunes are overtopped by the waves) and inundation regime (storm flooding submerges the coastal barriers)^7^. Catastrophic storm surge flooding is mainly associated with inundation regime. The susceptibility of a given coast to storm surge flooding may change in time due to several factors, among which are sea-level rise, storminess, as well as coastal landform development associated with short-timescale sediment supply changes^1,8,9^. However, knowledge of the role of these controlling factors is limited and can be assessed only if changes in other factors, such as sediment budget changes, tides, bathymetry, and wave fetch length, can be accounted for.

The instrumental and historical records go back only few hundred years, and thus the sedimentary archives provide important records of past coastal flooding. Moreover, comparison of recent, instrumentally recorded storms with historical and geological archives, reveals that they may not be an adequate reference to worst case scenarios; it is the sedimentary archive that may provide record long enough to capture the high magnitude and low probability events^10^.

The Baltic Sea is an European marginal sea connected to the North Atlantic Ocean. The sea is a non-tidal sea, where the maximum tidal height evaluated for a 100-year period at its southern coast in Poland is only 8 cm^11^. Due to the low tidal regime storm flooding signals are not influenced by tidal effects and major water level fluctuations are almost exclusively related to storm surges^10,12,13^ and well-documented past sea-level changes, thus making the Baltic Sea coastline suitable for palaeotempestological studies. The Baltic Sea area is characterized by a transitional oceanic-continental temperate climate that is sensitive to latitudinal shifts in North Atlantic Oscillation (NAO) and changes in westerly storm tracks^14^.

**Figure 1:**
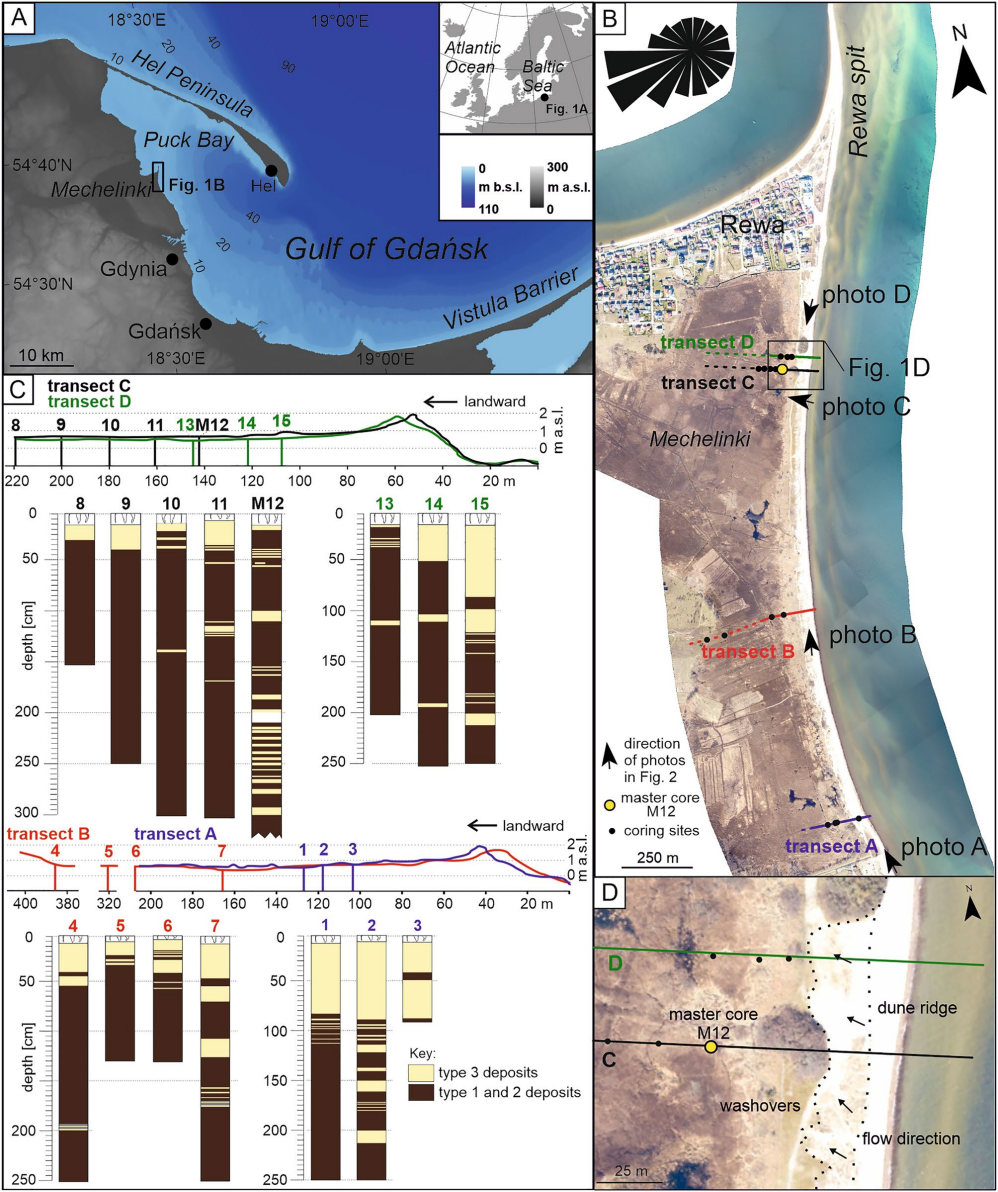
Location of the research area. (**A**) Mechelinki research area at the southern Baltic Sea coast (Poland), Puck Bay within the Gulf of Gdańsk. (**B**) Orthophoto map of the Mechelinki area with locations of coring sites and geological cross-sections. (**C**) Geological cross-sections through the depositional succession at Mechelinki, with deposits of type 1–3. (**D**) Close-up of recent wash-over fans (landward limit marked with dotted line). Bathymetric data: EMODnet service, resolution115 m; DEM: European Environmental Agency, hybrid model based on SRTM and ASTER GDEM with a spatial resolution of 30 m. Windrose predicting frequency of wind directions: https://www.meteoblue.com. Figure was created with CorelDRAW Graphics Suite 2019 (Corel Corporation: https://www.corel.com/en/old-versions/coreldraw-2019).

This study documents sedimentary succession from the Polish coast located at Mechelinki coastal wetland, Puck Bay within the Gulf of Gdańsk (Fig. 1). The main aims of the research are: (i) to reconstruct past extreme storm surge floodings and (ii) to assess susceptibility of the coastal area to past storm induced flooding, as a result of the combined effect of increased storminess, sea-level change and specific coastal morphology, in particular the development of a coastal barrier.

### Regional setting

**Figure 2:**
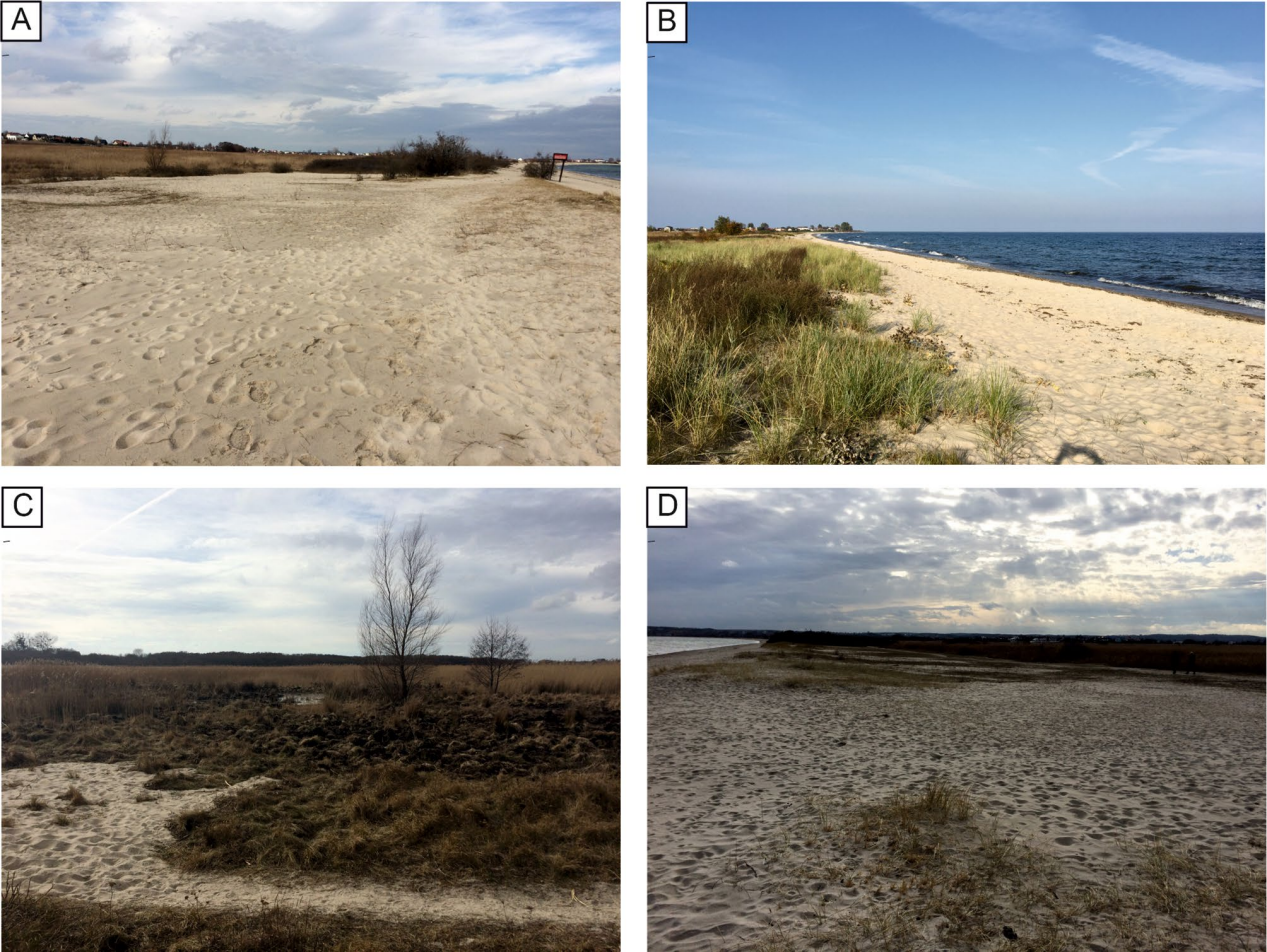
Photos of the Mechelinki research area (photo: K. Leszczyńska – all). (**A**) View to the north with recent washover fan (to the left) entering the wetland**. **(**B**) View to the north with the modern beach and foredune up to 2 m high. (**C**) View to the east, landward with partly stabilized foredune and wetland behind. (**D**) View to the south with the modern beach and foredune up to 2 m high. For locations of the photos see Fig. 1.

The Mechelinki coastal wetland is located on the coast of Puck Bay within the Gulf of Gdańsk, southern Baltic Sea, in Poland (Fig. 1). It represents a wetland reaching 0.8 m above mean sea-level frequently covered by water and shallow ground water level (Figs. 1, 2). The wetland developed from a lake formed after the decay of the Scandinavian ice sheet. Holocene lake and wetland sediments, including sandy intercalations, are underlain by Pleistocene glacio-fluvial and fluvial gravel and sands^15^. During the Holocene sea-level rise and associated transgression the Baltic Sea coastline gradually approached the lake. The Gulf of Gdańsk and Puck Bay witnessed the rapid transgression approximately 6.2 ka BP, during the last phase of the Littorina transgression^16^. The rates of sea-level rise decelerated after 5.8 ka BP from 3.6 mm/year to average rate of 0.4 mm/year from 4.5 ka BP until the Little Ice Age (LIA)^17^. Sea level was stable or slowly decreasing during the LIA (0.4–0.14 ka BP). During the past 140 years sea level rise increased to a rate of 1.0 mm/year until 1960 CE and accelerating to the present rate of 2.4 mm/year in Gdańsk and 2.0 mm/year in Władysławowo^18^. The modern net vertical crustal movements are close to zero^19^.

The shallow and brackish Puck Bay is the northwestern most extension of the Gulf of Gdańsk (Fig. 1), which is separated from the open sea by the barrier spit (Hel Peninsula). The average water salinity is approximately 7 PSU^20^. The study site at Mechelinki wetland is separated from the bay by a 34 to 45 m wide beach and beach ridge with foredune on top reaching a height up to 2 m above mean sea level (amsl). Washover fans are observed landward of the foredune crest (Fig. 1). Offshore, the seafloor is gently deepening, reaching a water depth of 10 m around 3 km from the shore.

Small rivers existing to the north of Mechelinki are characterized by very low flow levels of maximum 5 m/s and drain meadows associated with more than 5 m thick peat successions and supply negligible amounts of sediments to the bay. Thus, the sediments to the coastal system are supplied mainly by longshore and/or cross-shore transport.

Shallow water depths in Puck Bay result in lower, but more prolonged rise of water level during storms compared to the open coast, in particular in the case of the seiche effect. The mean decadal number of storm surges from 1960 to 2012 that exceeded 70 cm amsl, ranged from 156 to 253 events measured at the tide gauges of Hel and Gdańsk, respectively^21,22^. The theoretical 100-year maximum storm surge level is 180 cm amsl and exceeds the maximum measured sea level at the Gdańsk tide gauge station of 144 cm amsl during a storm in November 2004^22^. However, this storm surge did not cause extensive coastal flooding and recognizable sedimentary records in coastal wetlands^10^.

The majority of storm-weather conditions occurs in autumn and winter seasons. A low-pressure anomaly in the north, over Iceland, and a high pressure anomaly in the south over Azores, creates a steep pressure gradient enhancing the strength of westerlies influencing the Baltic Sea region^14^, in particular during positive NAO phases. Favorable conditions for extreme storm surge flooding in Puck Bay are created by the combination of water set-up at the eastern Baltic coast by persistent westerlies, subsequent seiche effect combined with wave refraction from the opposite coast of Gulf of Gdańsk and turning wind directions to northerly and easterly winds^22^. An extreme inundation event of this type occurred in the western Baltic Sea in November 1872 AD^23^.

Winds from northerly and easterly directions account for approximately 25% of total wind directions. The northerly wind speed of 15 m/s causes a wave height of approximately 0.2 m and easterly winds of up to 0.4 m^22^. North Atlantic storminess is defined in terms of the number of days with wind speeds exceeding 13.9 m/s (7 on the Beaufort scale), for one or more measurements during a 6 h period over the course of any single day^24^. In the Gulf of Gdańsk wind speeds above 15 m/s occur for 6 days/year on average. They are usually combined with the seiche effect and result in moderate waves between 0.2 and 0.4 m, depending on the direction of the wind^21^.

## Results

### Sedimentary units

**Figure 3:**
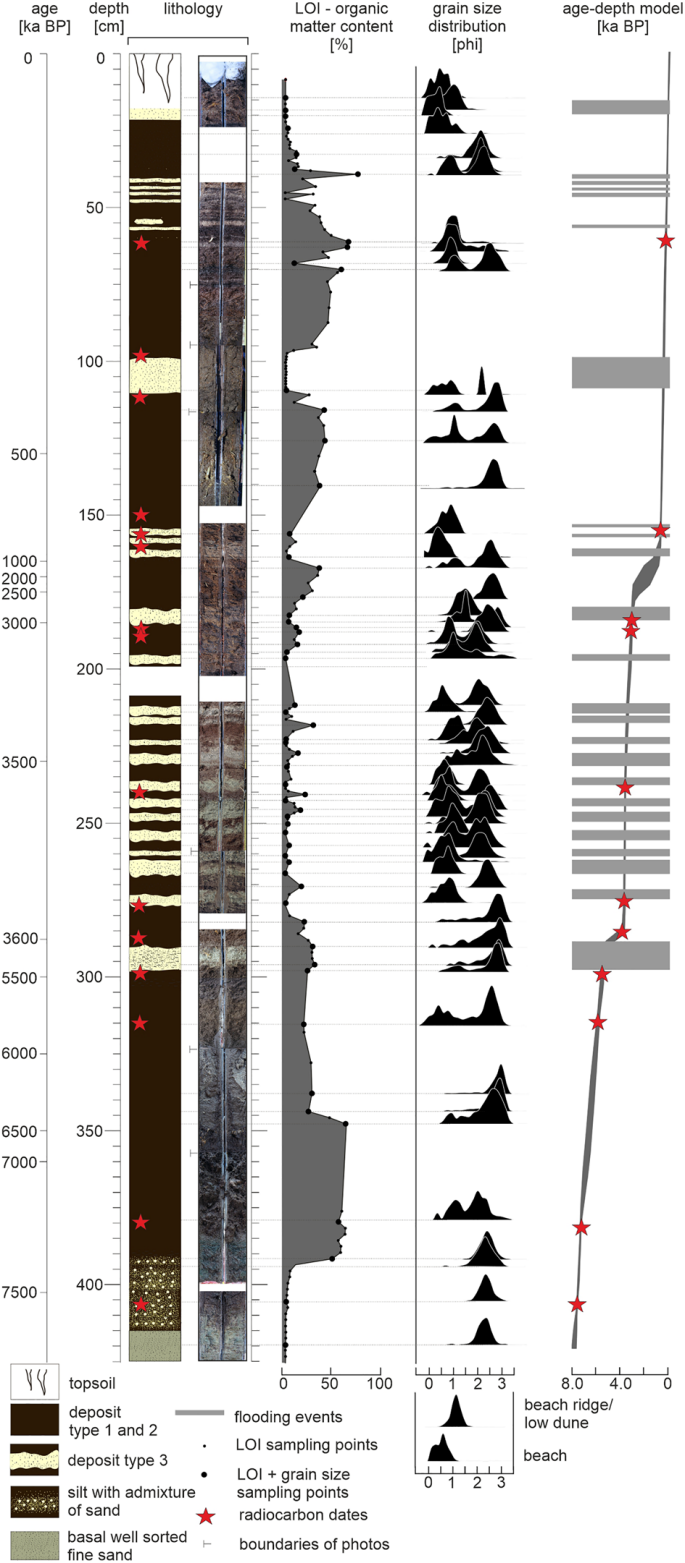
Master core M12. The core log, photography of the core, percentage of organic matter content, grain size distributions and age depth model. Depth scale was corrected for the mechanical compaction rate. See Fig. 1B for location of master core M12. Figure was created with CorelDRAW Graphics Suite 2019 (Corel Corporation: https://www.corel.com/en/old-versions/coreldraw-2019).

A detailed description of the sediment succession at Mechelinki is based on core M12 (Figs. 1, 3), which depths were corrected for mechanical compaction. The base of the core (425–415 cm) consists of well-sorted, unimodal, fine-grained sand of pale yellow color, which changes into massive, mottled gray and blue, silt with admixture of fine-grained sand (415–390 cm depth). Above, the succession consists of three intercalated sediment types (Table 1, Supplementary Table 4, Supplementary Fig. 1).

**Table 1:** Characteristics of various types of deposits from the mastercore M12.

Unit	Thickness in cm	% of Mineral matter based on LOI analysis (%)	Grain size distribution, mean grain size	Elements in which the particular type of sediment is enriched
Type 1	Few to few tens of cm	< 40–80	Bimodal, fine sand coarse sand	Mn, Fe, S, Zn, As
Type 2	Few to 10 cm	80–90	Bimodal, fine sand coarse sand	mixed Mn, Fe, S, Na, K, Rb, Zn, As
Type 3	Few cm	> 90	Unimodal, coarse sand	Na, K, Rb, Zn, As

The type 1 sediment comprises decomposed organic matter mixed with silt, clay and very fine sand. These sediments contain between 40 and 80% of mineral matter, which is more than within the type 1 sediments. The layers of sediment type 2 are often sandwiched between layers of sediment type 3. Close to the boundary with deposits of type 3, there are coarse-grained sands embedded within the matrix composed of organic matter, clay and silt.

**Figure 4:**
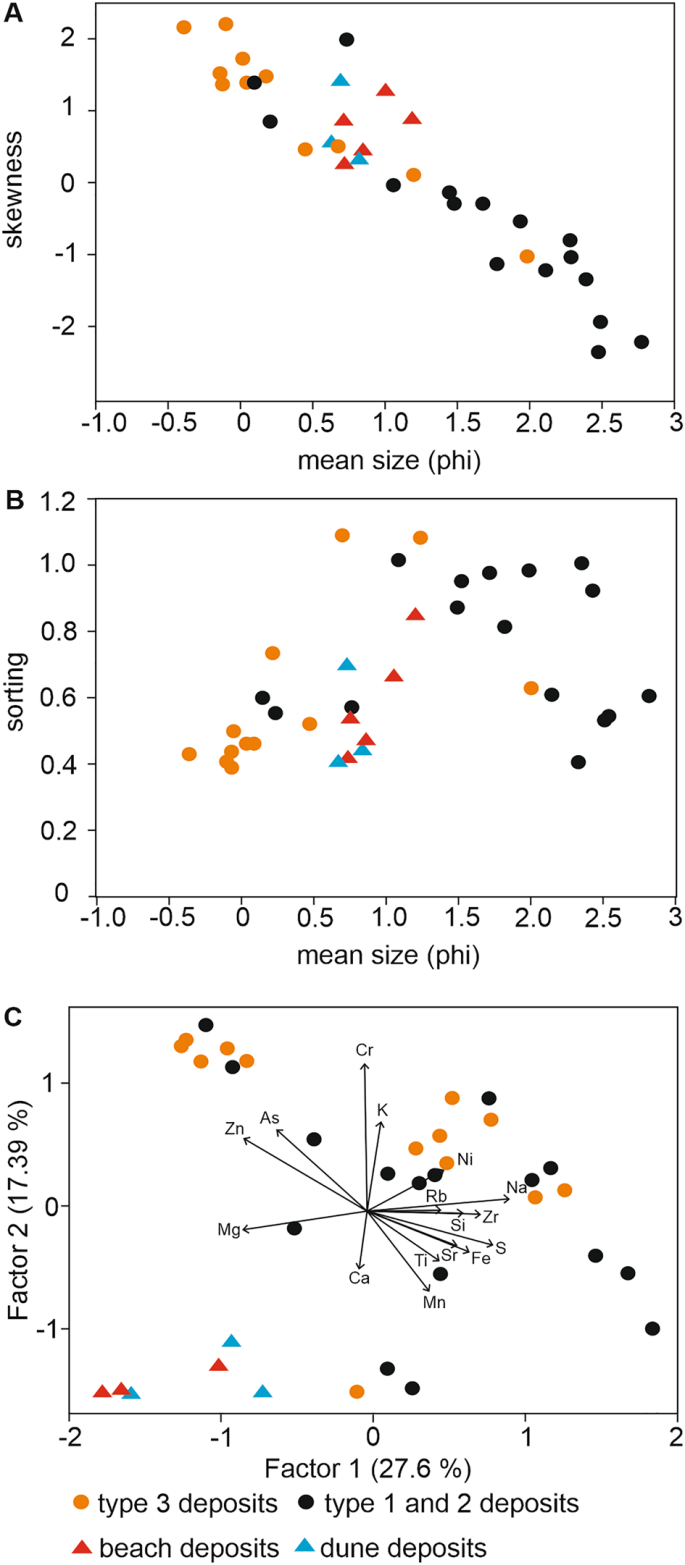
Grain-size and geochemical data. Samples were taken from all three types of sediments from the master core M12 from Mechelinki and modern beach and dune deposits. (**A**) Mean grain-size (in phi units) vs. skewness. (**B**) Mean grain-size (in phi units) vs. sorting. (**C**) Factor analysis of the geochemical data. The arrows accompanied by element symbols indicate the eigenvector values in the biplot.

Factor analysis reveals that sediment types 1 and 2 are clustered together in a single group termed thereafter wetland deposits. Their geochemical analysis indicates that they are enriched in Mn, Fe and S in comparison to deposits of type 3, as well as to beach and dune sediments (Fig. 4, Supplementary Table 3).

Sediments of types 1 and 2 (wetland deposits) are intercalated with sediments of type 3, which form 24, up to 10 cm thick, easily distinguishable, layers of pale to yellow sand (Fig. 3). All sand layers comprise > 90% mineral matter (dry mass), reveal an unimodal grain-size distribution and are classified as coarse sand (Fig. 3). Micromorphological analysis of sand layers reveals their clast-supported texture and inclined stratification (Supplementary Table 4, Supplementary Fig. 1). In some of the layers, decomposed organic matter fills the voids between skeletal sand particles. The lower contact of type 3 sediment layers is sharp and undulating and indicates erosion. Clasts of underlying organic matter rich deposits are incorporated into the overlying sand layers, as eroded from beneath rip-up clasts and as clusters of dispersed organic matter mixed with sand. Geochemical analysis shows that deposits of type 3 are enriched in Na, K and Rb. In samples from the uppermost section of the core there are also higher concentrations of Zn and As (Supplementary Table 3). However, the statistical analysis of the raw geochemical data does not allow to unequivocally separate the deposits grouped as the wetland sediments from those of the intercalated sand layers (Fig. 4).

The heavy mineral assemblage of the sand layers is dominated by amphibole, garnet and epidote with accessory amounts of other groups (Supplementary Table 6). In comparison to the reference dune sample, sand deposits within the core have higher amounts of amphibole and epidote and lower amounts of garnet.

The diatom counts reveal that the most common and abundant species were *Amphora pediculus*, *Cocconeis disculus*, *Karayevia clevei*, *Planothidium frequentissimu*, *Planothidium joursacense* and *Planothidium rostratum*. Despite the fact that diatom analysis was undertaken in order to characterized the environments of deposition of various types of sediment, diatom assemblages are similar within all the samples, regardless of the sediment type (Supplementary Table 5) indicating brackish to freshwater environments. Diatoms are very scarce in samples from depths of 16 and 106 cm.

The deposits of type 1–3 were traced in four geological transects (Fig. 1; transects A–D), the thickness of the sand layers composed of the type 3 deposits is irregular, their continuity cannot be determined. However, as a general rule all the sand layers are thicker closer to the sea-shore and thinner in landward direction. Apparent thinning seaward of some layers may be associated with the uneven surface on which the sand layer has been deposited. The layers below a depth of 100 cm from the ground surface pinch out closer to the coastline (between 60 and 160 m) than the upper layers (reaching between 140 and 320 m landwards). 

The core M12, selected as the mastercore for this study, represents the most complete record of storminess at that site. The lack of such a throughout evidence of marine coastal flooding at other coring sites reflects high discontinuity and patchiness of palaeotempestological depositional record. 

### Age control

The calibrated ^14^C dates of samples taken from master core M12 revealed an increase in age with depth, except for four samples excluded from the age-depth modeling (Supplementary Tables 7, 8). The age-depth model (Fig. 3, Supplementary Fig. 2) reveals three sections of relatively steady accumulation: 0–163 cm as compensated for mechanical compaction (0–150 cm as compensated for mechanical and natural compaction), accumulated since approximately 0.7 ka BP to present at a accretion rate of ~ 2.1 mm/yr; 181–291 cm (169–281 cm), accumulated between approximately 3.6 and 2.9 ka BP at an average rate of 1.4 mm/yr; and 298–390 cm (288–390 cm), accumulated between approximately 7.5 and 5.4 ka BP at a rate of 0.5 mm/yr. The natural compaction is assumed to be moderate due to the low content of organic matter. These three sections are separated by two intervals: 163–181 cm (150–169 cm) with very low rates of sediment accumulation, and 291–298 cm (281–288 cm) with deposition of just one sand layer.

## Discussion

### Origin of sand layers

There are multiple possible sources and depositional processes leading to sand layer formation within the lowland coastal succession. The sediment may come from the erosion of nearby dunes, beaches, older Pleistocene sands or offshore sediments. The Puck Bay represents semi-enclosed bay with no significant sediment supply from outside of the bay^25^. Moreover developed river system does not exist in vicinity of the research area. The depositional processes may include aeolian and beach processes as well as marine inundations (storm surge, tsunami, and sea-level rise). Below the characteristics of sand layers composed of type 3 deposits are compared with end-member samples (swash zone, lower beach, upper beach, dunes) as well as published results of offshore samples^26^ and are discussed to provide insight into sediment provenance and depositional processes.

Considering the general thinning landward thickness of discrete sand layers composed of type 3 deposits, it is likely that the sand was transported from the coast and the sea to the wetland. However, it is not possible to trace the lateral continuity neither in a cross-shore or longshore direction of the sand layers. Based on the mean grain size and the grain size distributions (Fig. 4a, b), the sand layers composed of the type 3 deposits are clearly distinguishable as coarser and unimodal in comparison to the wetland deposits comprising type 1 and 2 deposits. Type 2 sediments are different from type 1 in a way that they partly overlap in terms of grain size, with the deposits of type 3. This overlap is likely associated with very thin interlayering of type 2 and 3 sediments and mixing of material. Moreover, thin sandy layers of type 3 deposits may be completely incorporated and efficiently mixed with type 2 deposits, so macroscopic recognition is not possible^27,28^.

The comparison of type 3 sediments with end member samples from the beach and dune shows that there is an overlap of grain size characteristics, mainly the mean grain size with the samples from beach and dune. This in turn suggests that these two source areas could contribute to the pool of sediment sources. The alternative sources considered are offshore deposits. As compared with the published data^26^, the offshore component displays finer mean grain size that the type 3 deposits. In the closest vicinity of the Mechelinki site offshore sediments consist mainly of very fine (3–4 phi) to fine (2–3 phi) sand and only in the area to the north of the Puck Bay, along the Hel Peninsula, medium sized sand (1–2 phi) occurs.

The statistical analysis of the geochemical composition (Fig. 4c) reveals that all three major sediment types (both wetland deposits and sandy intercalations) overlap, however they are clearly distinguishable from modern beach and dune deposits (Fig. 4). Commonly used geochemical marine origin indicators are of limited significance in deciphering the origin of deposits at Mechelinki as the Puck Bay waters are brackish (ca. 7 PSU)^20^, therefore does not imprint a distinctive marine signal. Nevertheless, the core sections with sand layers have slightly higher concentrations of salinity indicators such as Na and K, both in sandy sediment type 3 and intercalated type 2. This is due to marine waters being involved in deposition of sediment type 3, and percolating downward into underlying deposits^29^ (Fig. 4c).

Within all samples analysed for diatoms species of wide ecological tolerance ranges and cosmopolitan character (*Amphora pediculus, Staurosira construens*) are present (Supplementary Table 5). The majority of taxa represent fresh/brackish to fresh-water organisms, characteristic of basic (*Karayevia clevei, Planothidium joursacense*) and highly conductive (*Planothidium rostratum, Pseudostaurosira brevistriata*), moderately to high trophy environment. Diatoms of identified taxa are benthic, associated with various types of sandy to epiphytic substratum. These results relate to previous study from the southern Baltic Sea coast^10,12^ as low salinity Baltic Sea is a habitat to marine, brackish as well as some freshwater species. Diatom assemblages do not differ significantly between type 1, 2 and 3 sediments. This in turn, may reflect influence of brackish Baltic Sea water on groundwater within the coastal meadows.

The heavy mineral assemblage of sand layers, although similar to dune and in particular beach sediments, also revealed some differences, suggesting potential contribution from alternative source, most likely from offshore. The detailed comparison of the heavy mineral data with the composition of offshore samples is precluded by the fact, that the concentrations of heavy minerals within the sediments of Puck Bay are very low^26^. However, in the light of established lack of fluvial component and semi-enclosed depositional character of the Puck Bay, the offshore component is identified as likely alternative source beside the beach deposits.

The combined evidence from micromorphology of the sediments, grain size, geochemistry and heavy minerals (Figs. 1, 3, 4, Supplementary Fig. 1, Supplementary Tables 1–6) indicates that sand layers are deposits from mixed sources, including beach, dune and nearshore sediments. Sharp (erosional in microscale) lower contacts with scouring features and rip-up clasts identified at the lower boundaries of sand units composed of type 3 sediments are typical characteristics of fast flow flooding events^12,30^ (Supplementary Fig. 1). Therefore, it is likely that sand layers intercalated with the wetland deposits were formed by storm surges or tsunamis.

The tsunami origin of sandy deposits at the southern Baltic sea coast was hypothesized earlier by Piotrowski et al.^12^. However, the sedimentary evidence of coastal flooding and geological evolution of the research area does not invoke the possibility of tsunami occurrence^10^. At present there is no universal set of diagnostic features allowing for easy identification of storm and tsunami deposits^31^. As a general rule, tsunami deposits extend hundreds to thousands of meters inland^32,33^. On the contrary, storm deposits are of significantly smaller lateral extent, usually limited to the zone relatively close to the beach^34^. The characteristic features present within tsunami deposits, which are absent or rare in storm deposits, are mud laminae and sedimentary indicators of seaward return flow^33,34^, both of them are not found within the studied event deposits at Mechelinki. Moreover, there is no established possible cause (seismic, volcanic, underwater landslide) of such a tsunami wave within periods mentioned nor there exists evidence of such an event in other nearby areas.

### The history of storm surge flooding

Here, we present the longest sedimentary record of storm surge flooding for the southern Baltic Sea to date; deposits of the last 7.5 ka BP include a record of coastal flooding in two periods: from approximately 3.6 to 2.9 ka BP and from approximately 0.7 ka BP until present.

**Figure 5:**
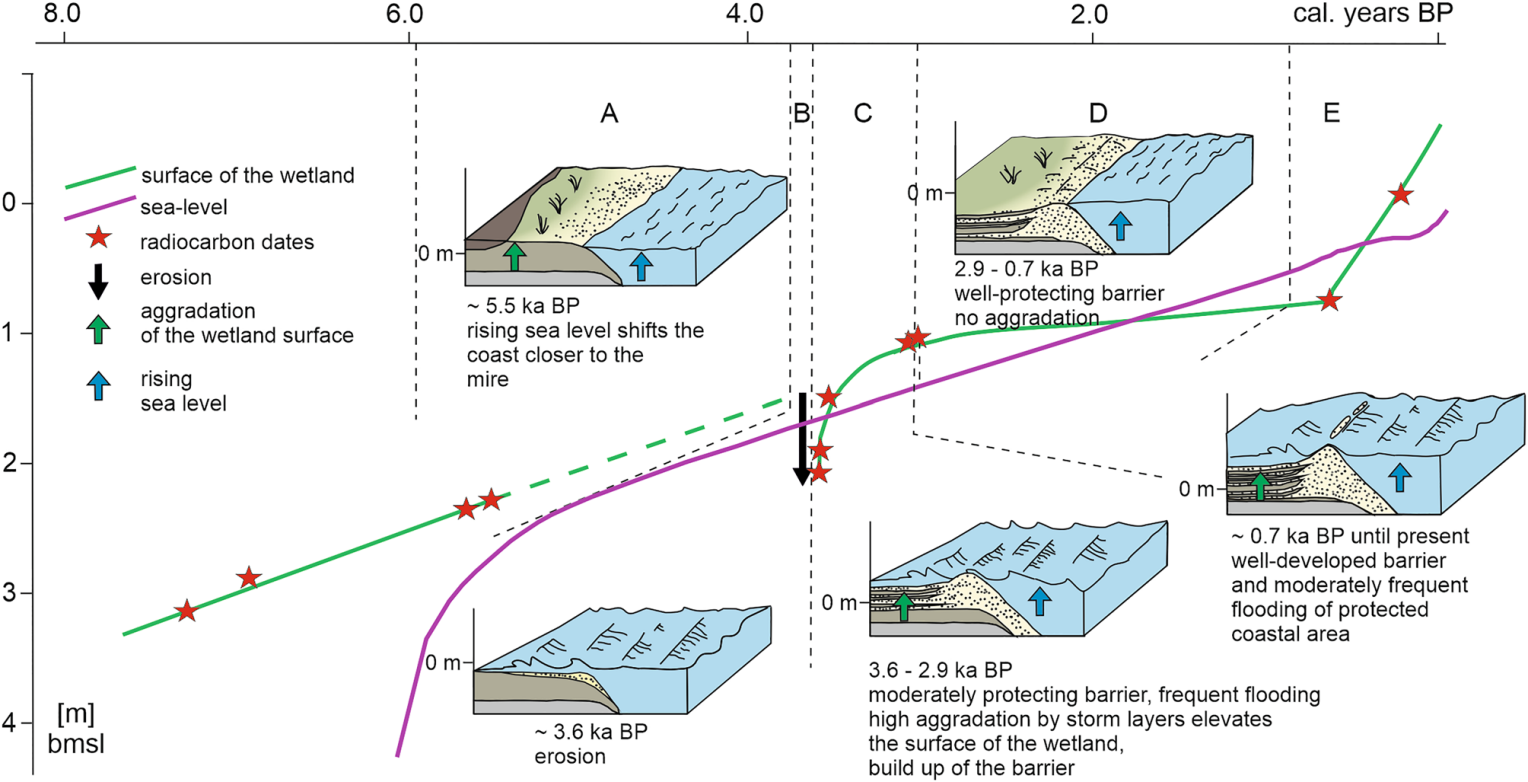
Sea level and coastal landform development reconstruction. Sections A to E depict discrete time spans represented schematically within the block-diagrams. The depths of age dating points indicated on the curve are corrected for mechanical and natural compaction. Figure was created with CorelDRAW Graphics Suite 2019 (Corel Corporation: https://www.corel.com/en/old-versions/coreldraw-2019).

Prior to the event at 3.6 ka BP the study site was distant from the shoreline. During the marine transgression, the shoreline approached the vicinity of the wetland that vertically aggregated following sea-level driven rise in groundwater level. Approximately 3.6 ka BP, the sea was 1.5 m lower than today, and, assuming the nearshore bathymetry gradient was similar to the present day, the shoreline was located approximately 200 m offshore of the modern one^35^. The new coastal morphology was formed at the site not affected previously by marine processes and the new coasts were not protected by extensive coastal landforms. Thus, the coast was particularly susceptible to erosion. Interpretation of the land surface elevation and sea-level reconstruction (Fig. 5) suggests that significant storm event(s) took place at that time and caused an erosional lowering of the wetland surface. It is not possible to assess in detail the scale of this lowering due to the uncertainty regarding the sea-level reconstruction, as well as the vertical growth and compaction of the wetland between 5.5 and 3.6 cal. ka BP (Fig. 5, green dashed line). From the core (Fig. 3) we estimate that the wetland surface above the first storm layer was at 2.1 below mean seal-level (bmsl), what would correspond to 0.6 m below the contemporary sea-level (Fig. 5). The following storms could easily inundate marginal parts of the wetland. The low slope of the shoreface favoring the wave skewness that pushed sand onshore promoted succeeding lateral deposition at the flat, low lying site. The sandy shoreline was partly restored after the storm events, but a massive beach ridge was unlikely to be developed due to juvenile character of the coastline and multiple subsequent storms at that time.

The erosion at the base of the storm layers at 3.6 ka BP could have also been explained by the transgressive retreat of the shoreline. As the shoreline retreated across the core site with rising sea-level the wetland was eroded. Then we must assume that the vertical accretion extended to a lateral progradation of the shoreline with deposition of wetland sediments intercalated with storm sand layers while the sea level was continuously rising. Ultimately, the sand deposited by storm coastal flooding stabilized the coast and protected it from subsequent flooding^9^.

The period between 3.6 ka BP and 2.9 ka BP represents the first well-preserved high-resolution record of at least 15 major storm surge flooding events, represented by sand layers (type 3 sediment). The long-term average recurrence time of the events was approximately 46 years, and the average frequency was approximately 2.1 per century. The storm layers are distributed evenly throughout this core section, although amalgamation of storm event layers cannot be excluded. Assuming that the shoreline was approximately 200 m offshore of the modern shoreline, we conclude that any possible storm induced flooding, archived within the core as sandy layer, must have had a minimum landward inundation distance of approximately 360 m (corresponding to the distance of the coring site from the shoreline at that time).

The period between 2.9 and 0.7 ka BP was possibly characterized by very low accumulation rates, likely caused by the low water table corresponding to regional droughts^36,37^, which limited the accretion of sediments in the drying wetland (Fig. 5). The shoreline approached the present day position, coastal landforms developed (Fig. 2), as the coastal zone accumulated some excess sediment due to local shoreline and nearshore erosion during the ongoing transgression. These coastal landforms provided a barrier that protected the wetland, which was 0.4 m bmsl at 0.7 ka BP, against extensive flooding. There is no evidence for substantial erosional event at the beginning of this younger period, however we cannot exclude the contribution of the erosion to the resulting low accumulation rate.

From 0.7 ka BP onwards, the accumulation rate significantly increased to reach approximately 2.1 mm/year. Although the surface of the wetland was initially below sea level, it was rarely inundated, being protected by well-developed coastal morphology/coastal barriers, i.e., (fore)dunes on top of beach ridges. The sedimentary record for this period includes 9 storm layers, which are thinner than those during the previous period (3.6–2.9 ka BP). The recurrence time of the events was approximately 77 years, and the frequency was approximately 1.3 per century. However, the storm surge layers are not distributed evenly, there is one relatively thick layer, which may correspond to major storm in 1497 AD^12^, however, most of the layers are concentrated in the core section dated to approximately 1750–1900 AD. The frequency of storm surge flooding for this period alone was 4.2 per century. If we assume the stable position of the shoreline, then the minimum inundation distance based on the extent of the sand layers within transect B was up to 320 m inland.

### Major controls on storm surge flooding

A high-resolution sedimentary archive from Mechelinki provides an opportunity to compare factors controlling extreme storm surge flooding, including sea-level change and the development of coastal landforms, inherently associated with the sediment budget^8,38,39^, within the two periods, each of them spanning approximately 700 years. The timing of enhanced storminess between 3.6 and 2.9 ka BP depicted from Mechelinki sedimentary archive fits into the broad scheme of Holocene storm periods for NW Europe based on Seine estuary sedimentary archive and Mont-Saint-Michel Bay in northern France^40^. There enhanced storminess is postulated for the period of 3.3 to 2.7 ka BP. Similarly this enhanced storminess period remains in agreement with the changing enhanced zonal North Atlantic storminess reconstruction based on wind-transported material in Filsø coastal wetland in western Denmark^41^, where enhanced storminess was suggested to occur between 3.3 and 2.4 ka BP. The discussed period also overlaps with periods of storm-related beach ridge formation periods in Estonia, eastern Baltic^42^.

**Figure 6:**
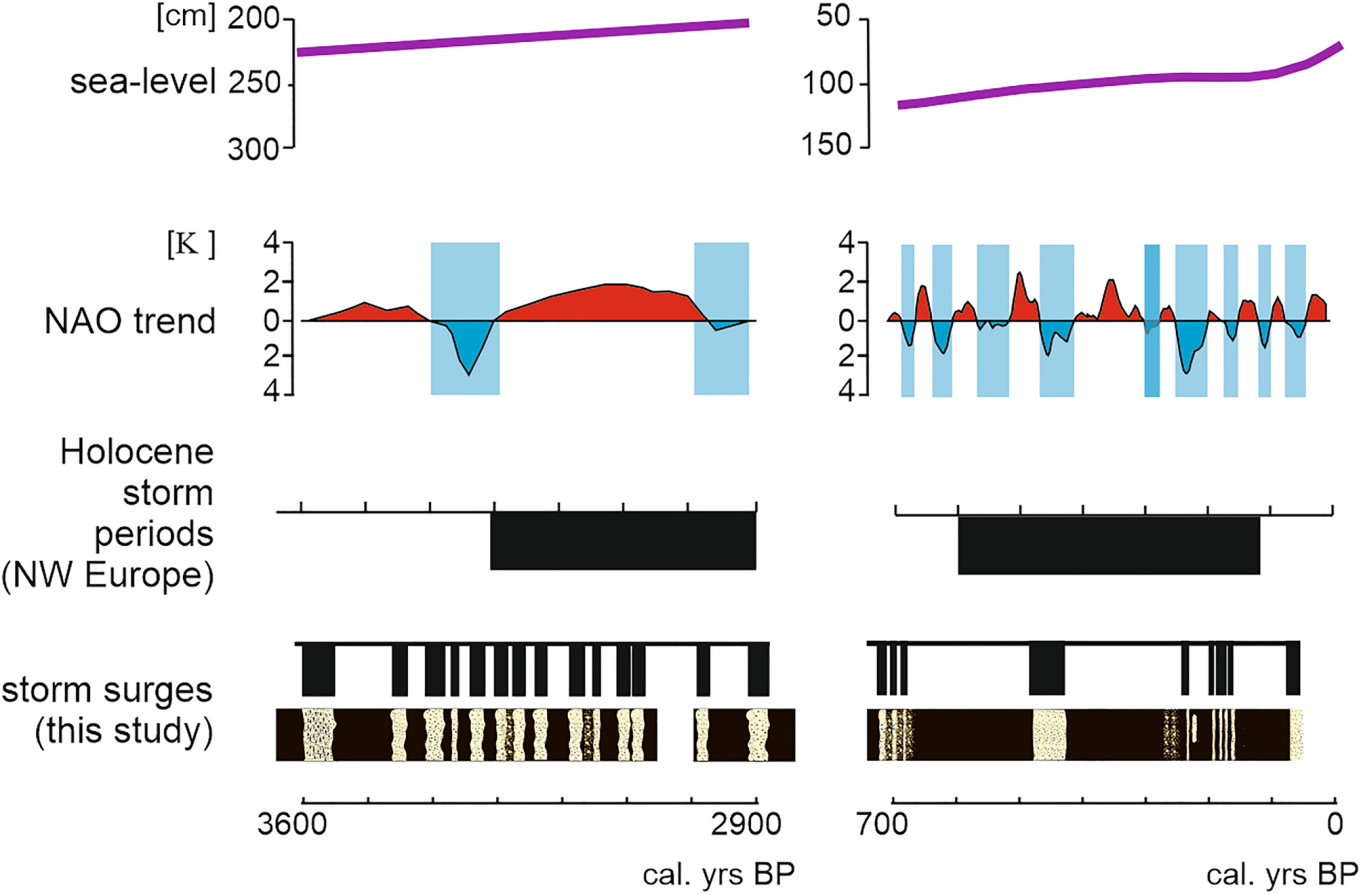
Comparison of the storm surge flooding controlling factors within the two studied time periods. Data sources: sea level trend based on^16,17^, NAO trend 3.6–2.9 ka BP based on^43^ and 0.7 ka BP until present based on^46^, Holocene storm periods in northwestern Europe based on^40^. Figure was created with CorelDRAW Graphics Suite 2019 (Corel Corporation: https://www.corel.com/en/old-versions/coreldraw-2019).

At Mechelinki storm surge flooding occurred during steadily rising sea level. The recorded storm flooding frequency was constant over the whole time span of 3.6 to 2.9 ka BP. This period was characterized by weakly positive NAO oscillation pattern, but including some extended periods of strong negative NAO states^43^ (Fig. 6).

During the second period from 0.7 ka BP until the present, the climatic conditions were more variable than during the first: the initial cooling was intensified during LIA and then followed by recent warming^43–45^ (Fig. 6). Before the LIA, between 950 and 1300 AD, the NAO remained mainly in a positive phase. From 1400 to 1800, during LIA, negative phases occurred more frequently^14^. NAO turned again to more positive phases from 1850 AD onward^46^. Similarly, the sea-level change trends were variable: initial steady rise, followed by a stabilization or even slight drop during the LIA and the most recent increase. Several sedimentary and historical records of storm surge flooding exist for the southern Baltic Sea coast for the last ca. 2 ka and revealed that the recorded events occurred almost exclusively in since the late fourteenth century to the onset of twentieth century^10,12^. The storm surge flooding frequency varied throughout this period: inundations were most frequent particularly during the LIA termination, when the NAO remained mostly in positive mode and when the sea level was almost stable.

During the older period the shoreline was approximately 200 m offshore of the modern one, and the studied wetland, accreted along with the sea-level rise^16,47^*.* However, increasing pressure from the transgressive sea, exposed it finally to storm-induced coastal flooding (Fig. 5). At that time (after 3.6 ka BP) the storm flooding events reached at least about 360 m inland. The relatively thick storm layers indicate the coastal landforms protection against flooding did not reach critical dimensions for full protection^48^ and the wetland was frequently flooded until 2.9 ka BP. Then, after the deposition of sand layers, the surface of the wetland was elevated and reduced the available accommodation space.

Analysis of the mastercore and the reconstructions of sea-level suggest that at the beginning of the second period (from 0.7 ka BP to present time) the surface of the wetland was below sea level, providing accommodation space for accumulation. Despite this, only the most severe storm surge events flooded the area. The likely reason is the development of protecting coastal barriers in form of the beach ridge with (fore)dune on its top, that could stabilize the shoreline and served as a defense against storm surge flooding until the transition to LIA. However, even during LIA the storms left thinner and less extensive sand layers than during the older studied period.

Our study indicates that archived evidence of intense storminess is simultaneous with enhanced storminess episodes identified elsewhere in the North Atlantic region^40^. These storminess periods are associated with shifting circulation patterns (especially NAO) and storm tracks ^49^. Despite the fact that both time spans represent periods of enhanced storminess recognized in northwestern Europe^40^ with the rising or fluctuating sea-level, the stage of coastal landforms development was different as well as the sedimentary record of storm flooding.

## Conclusion

The results of the current research add to ongoing discussion on the factors controlling storm induced coastal flooding. Sedimentary archives may be useful not only in deciphering the history and frequency of storm events, but also provide insights into the susceptibility of the coast to flooding. Puck Bay is an example of lowland sandy coast where due to negligible influence of tides, well known sea-level history and minor anthropogenic influence it was possible to perform critical assessment of the interplay of enhanced storminess, sea-level change, coastal landforms development and storm induced coastal flooding. At Mechelinki site periods of climatically driven high-frequency storm flooding took place during different sea-level change trends. One of the most important factors controlling the susceptibility of the coast to extreme storm surge flooding was the stage of evolution of costal landforms intrinsically associated with the sediment budget. Moreover the presented record revealed that the local coastal flood hazards is more extensive than the one reveled from instrumental record.

Our results may be considered to provide an outlook for the important role of coastal barriers in defending lowland coasts against storm surge flooding. Coastal barriers should be protected and preserved, their destruction due to natural processes or human action may lead to more frequent and severe flooding.

## Methods

To interpret the origin of sand layers and reconstruct coastal wetland environments, the deposits were dated (Fig. 3, Supplementary Tables 7, 8, Supplementary Fig. 2) and characterized through loss-on-ignition, grain size (Figs. 3, 4, Supplementary Tables 1, 2), geochemical (Fig. 4, Supplementary Table 3) and micromorphological analyses (Supplementary Table 4, Supplementary Fig. 1) with diatom and heavy mineral analyses used as supplementary methods (Supplementary Tables 5, 6). Moreover, several end member samples from the beach and dune were analyzed in the same way (Supplementary Table 9).

### Fieldwork

Initially, 15 Russian sampler cores were extracted along four transects in the north, middle and south of the research area (Figs. 1, 2). The exact location of the cores was determined by GPS (Garmin eTrex × 22), and their height was read from LIDAR data. LiDAR dataset and ortophotomap for the coastal area was supplied by the Polish Maritime Authority. Elevation data and aerial images were acquired 23–25.03.2020. The vertical data reference of LiDAR is PL-ECRF2007-NH. The original mean scanning density was approximately 2–12.4 points per m^2^ with the mean spatial error of 0.02 m and mean vertical error of 0.07 m. The spatial resolution of ortophotomap is 0.1 m with a mean error of 0.15 m. Morphologic profiles that were used to construct geological sections based on the LiDAR dataset after applying a moving average to surface elevation to smooth the individual profiles' curve within the meadow terrain.

Soil sampler cores were described in the field. Master core M12 was taken using a Cobra TT mechanical drill. The compaction rate within core M12 was calculated based on the penetration depth compiled with the length of the core. Mechanical compaction was calculated in the field and taken as the depth scale for the core description. Natural compaction was estimated for organic-rich sediments by 10–35% in thickness according to depth. No compaction was assumed for sand layers of sediment type 3. Calculated compaction rates aid the reconstruction of the land surface curve in Fig. 5.

Master core M12 was described in detail and sampled for further analyses. Laboratory analyses were also performed on 8 end member samples taken from the swash zone (2 samples: SZ1, SZ2), lower beach (2 samples: LB1, LB2), upper beach (1 sample: UB1) and beach ridge and initial dunes (3 samples: D1, D2, D3). Their coordinates are given in Supplementary Table 9.

### Geochronology

The chronology of master core M12 was based on accelerated mass spectrometry (AMS) ^14^C (Supplementary Fig. 2, Supplementary Tables 7, 8). Fourteen bulk peat samples and a single wood sample were selected for radiocarbon dating to provide a general core chronology and to date particular event layers. In the latter case, the samples were collected from below and above selected sandy layers. The samples were measured in the Poznań Radiocarbon Laboratory (Poland), Beta Analytics Laboratory (USA) and GADAM Centre (Gliwice, Poland). Radiocarbon ages were calibrated with CALIB Rev. 8.1.0^50^ using the IntCal20 calibration dataset^51^. The combined age-depth model for core M12 was constructed using the available results and applying the Bacon software package (Supplementary Fig. 2)^52^. The sandy layers were treated as single event deposits, and the 4 ages were not considered, as they provided significantly older ages than samples taken from sediments below them.

### Physical core description, loss-on-ignition and grain-size analyses

Master core M12 was photographed and described in the laboratory in terms of physical appearance, namely, sediment type, texture, color, and the type of boundary between identified sediment types. The loss-on-ignition technique, performed on 185 samples, was used to establish the percentage amount of organic versus mineral matter within the deposits^53^ (Fig. 3, Supplementary Table 1).

Sediment grain size was measured (Fig. 3, Supplementary Table 2) on 60 samples from core M12 and 8 end member samples: two from the swash zone SZ1, SZ2, two from the lower beach LB1, LB2, one from the upper beach UB1, and three from the beach ridge and initial dunes D1, D2, D3 (coordinates in Supplementary Table 9). Morphology G3 optical automated microscope was used. The analysis was undertaken at the Faculty of Geographical and Geological Sciences, Adam Mickiewicz University in Poznań, Poland. Prior to analysis, the samples were burned in a muffle furnace at 550 °C for 3 h to remove organic material. As the amount of siliciclastic material smaller than 63 µm was negligible prior to the analysis on Morphology G3, samples were wet sieved on a 63 µm sieve. The grain-size distribution was plotted with Gradistat 8.0, and grain size statistics were calculated using the logarithmic method of moments^54^.

### Micromorphology

Selected sections of master core M12, comprising sandy layers, were subjected to micromorphological analysis (Supplementary Table 4, Supplementary Fig. 1). The deposits extracted from the coring tube were dried and impregnated using epoxy resin. After impregnation, the samples were cut into thin sections. Slides were scanned on a high-resolution scanner to produce images of all samples and analyzed under a petrographic microscope under plane and cross-polarized light. The description protocol followed Leszczyńska et al.^55^ and the terminology developed by pedologists^56^. Particular attention was given to depositional and erosional structures typical for flood deposits^34^.

### Geochemistry

A handheld X-ray Tracer III ED-XRF (Bruker AXS, USA) spectrometer was used to assess the concentrations of 9 selected major and 7 selected trace elements in 109 samples from master core M12 and 6 end member samples: from the swash zone SZ1, from the lower beach LB1, from the upper beach UB1, and three from the beach ridge and initial dunes D1, D2, D3 (Supplementary Table 3, coordinates of end member samples in Supplementary Table 9). The spectrometer was operated in the quantitative mode described by the manufacturer. Two calibration standards were used: Bruker Mudrock Major for analysis of Ca, Ti, S, Si, Na, K, Cr, Zn and Mg and Bruker Mudrock Trace for determination of Mn, Sr, Fe, Zr, Rb, Ni, and As^57^. Geochemical analysis included major and trace elements, e.g., Mg, Ca, Na, K, and Rb, which are considered to be indicators of marine origin.

Factor analysis was undertaken on 29 samples from core M12 and 6 end member samples (3 beach and 3 dune samples) for which geochemical analyses were available for all the elements. The calculations were made in R software^60^ using 2 factors and function “factanal” from package “stats”. Function “factanal” performs maximum-likelihood factor analysis on a covariance matrix or data matrix. Following arguments of the function were used: scores = ”Bartlett”, rotation = varimax. To plot the results of the factor analysis function “autoplot” was used. The aim was to establish the similarities between the discrete samples.

### Diatom analysis

The diatom analysis was conducted for selected sandy event layers on 9 samples; in one case, the organic deposits below and above the minerogenic layer were also analyzed (Supplementary Table 5). Preparation of collected material for diatom analysis was conducted according to standard procedures^58^. Diatom valves were identified to the lowest possible taxonomic level with reference to^59^.

### Heavy minerals analysis

The heavy minerals were analyzed from 4 samples from the sand layers within master core M12 and 2 end member samples: from the beach LB1 and from the dune D2 (Supplementary Table 6, coordinates of end member samples in Supplementary Table 9). Samples were dried and sieved, and minerals from fractions of 125–250 µm were separated using sodium polytungstate liquid of density < 2.85 g/cm^3^. Microscopic slides were prepared using Canada Balsam and analyzed under a petrographic microscope with cross-polarized optics.

## Supplementary Information

Supplementary Information.

## Data Availability

All the data and materials are available on UAM OneDerive cloud service.
