# Supplementary material for: Controls on coastal flooding in the southern Baltic Sea revealed from the late Holocene sedimentary records
Source: Sci Rep. 2022 Jun 11;12:9710. doi: 10.1038/s41598-022-13860-4 (PMC9188548; doi:10.1038/s41598-022-13860-4)
Supplement: Supplementary file 1 — Supplementary Information. [file 41598_2022_13860_MOESM1_ESM.pdf]

Title: Controls on coastal flooding in the southern Baltic Sea revealed from the late Holocene sedimentary records

Short title: Controls on storm surge flooding

Authors

Karolina Leszczyńska[1]*(0000-0001-8729-1733), Karl Stattegger[1] (0000-0001-5104-2030), Damian Moskalewicz[2] (0000-0002-3248-1860), Robert Jagodziński[1] (0000-0002-8469-215X), Mikołaj Kokociński[3] (0000-0002-0552-9769), Przemysław Niedzielski [4] (0000-0002-2787-9057), Witold Szczuciński[1] (0000-0003-2466-2263)

Affiliations

[1] Geohazards Research Unit, Institute of Geology, Adam Mickiewicz University, Bogumiła Krygowskiego 12, 61-680 Poznań, Poland, e-mail address: karles3@amu.edu.pl.

[2] Department of Geomorphology and Quaternary Geology, University of Gdańsk, Bażyńskiego 4, 80-952 Gdańsk, Poland.

[3] Hydrobiology Department, Faculty of Biology, Adam Mickiewicz University, Uniwersytetu Poznańskiego 6, 61-614 Poznań, Poland.

[4] Faculty of Chemistry, Adam Mickiewicz University, Uniwersytetu Poznańskiego 8, 61-614 Poznań, Poland.

SUPPLEMENTARY MATERIALS

Supplementary Table 1: Loss-on-ignition (LOI) analysis results for samples from master core M12 .

Depth [cm]	% of LO	% of mineral fraction
8	1.00	99.00
10	20.68	79.32
12	0.77	99.23
14	0.89	99.11
16	0.48	99.52
18	0.92	99.08
20	0.79	99.21
22	0.99	99.01
24	4.38	95.62
25.5	4.00	96.00
26.5	2.10	97.90
27.5	4.30	95.70
28.5	7.52	92.48
29.5	6.71	93.29
30.5	6.86	93.14
31.5	15.76	84.24
32.5	15.91	84.09
33.5	15.52	84.48
34.5	5.10	94.90
35.5	17.36	82.64
36.5	18.53	81.47
37.25	13.47	86.53
37.75	35.29	64.71
39	97.91	2.09
41	5.34	94.66
43	7.51	92.49
44.5	2.19	97.81
45.5	4.94	95.06
47	1.95	98.05
49	40.74	59.26
51	34.24	65.76
53	47.80	52.20
55	49.56	50.44
57	2.54	97.46
59	63.43	36.57
61	88.22	11.78
62.75	86.27	13.73
64.5	52.26	47.74
66	60.44	39.56
68	12.95	87.05
70	77.83	22.17
71	72.22	27.78
74	58.58	41.42
77.5	62.75	37.25
82.5	60.65	39.35
87.5	59.02	40.98
94.5	36.79	63.21
95.5	43.92	56.08
96.5	11.79	88.21
97.5	2.40	97.60
98.5	2.08	97.92
99.5	1.88	98.12
100.5	1.20	98.80

101.5	1.41	98.59
102.5	1.37	98.63
103.5	1.91	98.09
104.5	1.38	98.62
105.5	1.33	98.67
106.5	1.07	98.93
107.5	1.24	98.76
108.5	1.34	98.66
109.5	2.02	97.98
111	33.42	66.58
113.5	12.71	87.29
116	54.23	45.77
118.5	46.30	53.70
121	53.48	46.52
126	55.04	44.96
131	47.04	52.96
136	41.28	58.72
141	48.15	51.85
157	7.00	93.00
159	14.66	85.34
160.5	6.94	93.06
162	2.17	97.83
164.5	5.07	94.93
167.5	47.10	52.90
170	45.03	54.97
172.5	32.06	67.94
175	37.40	62.60
177	24.88	75.12
179	13.37	86.63
181	16.48	83.52
183	5.91	94.09
185	5.07	94.93
187	15.56	84.44
188.5	19.34	80.66
191	12.98	87.02
192.5	17.36	82.64
195	2.66	97.34
197	0.93	99.07
212.5	13.86	86.14
213.5	6.10	93.90
214.75	1.31	98.69
216	9.44	90.56
217	1.55	98.45
219	38.88	61.12
221	11.54	88.46
222.5	5.05	94.95
223.5	1.78	98.22
224.5	0.93	99.07
225.5	1.88	98.12
226.75	6.08	93.92
228	18.28	81.72
229	10.36	89.64
230.5	4.25	95.75
231.5	5.62	94.38
232.5	2.67	97.33
234	5.43	94.57
236.5	8.55	91.45
237.5	0.99	99.01
238.5	0.92	99.08
239.5	0.83	99.17
240.5	4.52	95.48

241.5	27.82	72.18
242.5	2.10	97.90
243.5	1.05	98.95
244.5	12.61	87.39
245.5	13.82	86.18
246.5	21.37	78.63
247.5	12.76	87.24
248.5	3.78	96.22
249.5	3.67	96.33
251	4.00	96.00
254	0.60	99.40
258	5.79	94.21
261.5	0.68	99.32
263.5	5.64	94.36
267	0.45	99.55
271.5	22.69	77.31
274	5.87	94.13
277.5	0.43	99.57
281	6.46	93.54
283	26.74	73.26
285	26.48	73.52
287	18.78	81.22
289	3.34	96.66
291	38.32	61.68
293	37.51	62.49
295	37.78	62.22
297	40.90	59.10
299	31.15	68.85
316.5	25.95	74.05
319	26.50	73.50
329	35.80	64.20
339	37.38	62.62
345	32.31	67.69
347	61.19	38.81
349	84.57	15.43
377.5	78.23	21.77
381	74.00	26.00
383	83.45	16.55
385	82.75	17.25
387	73.07	26.93
389	77.11	22.89
391	76.72	23.28
393	65.03	34.97
395	14.78	85.22
397	7.31	92.69
399	6.61	93.39
401	4.20	95.80
403	3.36	96.64
405	2.73	97.27
407	1.96	98.04
409	4.33	95.67
411	1.28	98.72
413	1.34	98.66
415	0.88	99.12
417	1.11	98.89
419	1.07	98.93
421	0.91	99.09
423	0.68	99.32
425	0.79	99.21
427	0.66	99.34
429	0.67	99.33

431	0.89	99.11
433	0.50	99.50
435	0.61	99.39
437	0.48	99.52
439	0.56	99.44
441	0.60	99.40
443	0.40	99.60
445	0.37	99.63
447	0.43	99.57
449	0.60	99.40

Supplementary Table 2: The grain size statistics of the analyzed samples from the master core M12 (sample names are presented by sediment depth) and end member samples.

Depth [cm]	Mean [phi]	Sorting	Skewness
8	-0.093	0.411	1.568
12	-0.056	0.489	1.469
16	0.425	0.514	0.285
18	-0.379	0.433	2.159
21.5	0.131	0.598	1.411
32.5	2.130	0.611	-1.224
37.35	2.306	0.409	-0.784
39	1.700	0.969	-0.281
60	0.741	0.569	1.981
62.75	0.954	0.864	1.083
68	0.447	0.447	2.685
70	1.960	0.984	-0.545
111	0.680	1.089	0.501
116	2.236	1.047	-0.978
126	1.473	1.260	-0.102
141	2.731	0.538	-2.132
157.5	0.229	0.573	1.693
164.5	-0.141	0.748	2.808
167.5	1.909	1.037	-0.827
177	2.666	0.410	-0.706
183	2.277	0.455	0.017
185	2.152	0.404	-0.924
188.5	2.320	0.434	-0.219
192.5	2.381	0.573	-1.845
195	0.979	0.882	0.096
197	1.226	0.768	-0.365
199.5	0.945	1.018	0.828
212.5	1.872	0.578	-0.997
214.75	0.037	0.529	1.816
219	2.361	0.511	-1.001
223.5	0.696	0.514	0.505
224.5	1.734	0.648	-0.198
228	2.159	0.584	-0.719
232.5	1.279	0.751	-0.172
238.5	-0.093	0.433	2.206
241.5	1.077	1.014	-0.022
243.5	0.075	0.466	1.404
246.5	2.513	0.540	-1.916
248.5	1.216	1.085	0.103
251	1.789	0.816	-1.126
254	0.457	0.517	0.478
258	1.492	0.955	-0.281
261.5	1.985	0.630	-1.014
263.5	0.194	0.733	1.476
267	0.031	0.463	1.723
271.5	2.489	0.529	-2.326
277.5	0.220	0.554	0.852
283	2.404	0.923	-1.339
291	2.298	0.998	-1.018
297	2.576	0.778	-1.199
299	2.319	1.004	-1.030
316.5	1.732	1.224	-0.387
339	2.792	0.605	-2.208
345	2.744	0.640	-1.949
349	2.199	0.942	-0.849
381	1.466	0.879	-0.114
393	2.353	0.405	-0.169
395	2.337	0.506	-0.668
407	2.399	0.354	0.175
421	2.159	0.451	-1.079
beach	0.845	0.472	0.395
beach	0.74	0.542	0.307
beach	1.195	0.847	0.903
beach	0.723	0.419	0.9
beach	1.029	0.664	1.286
dune	0.654	0.405	0.549
dune	0.716	0.692	1.456
dune	0.824	0.442	0.348

Supplementary Table 3: Geochemistry of samples from master core M12 (sample name is given by sediment depth) and end member samples.

depth	Ca	Ti	S	Si	Na	K	Cr	Zn	Mg	Mn	Sr	Fe	Zr	Rb	Ni	As
[cm]	[mg kg-1]															
6.5	3524	175	2497	119814	2930	2512	67	80	78515	207	42	6308	35	57	12	4
7.5	6425	351	3615	279661	3234	9662	155	181	<1	169	7	5005	1	45	13	9
8.5	6698	459	4166	192068	2937	12078	172	204	41516	161	23	5290	11	45	11	8
9.5	5543	840	5388	158609	2554	15041	168	164	54263	172	27	6367	43	49	14	7
10.5	4647	448	5015	<1	899	13326	187	181	127464	152	15	5152	11	49	13	10
11.5	3429	453	4109	8618	2271	12655	175	162	64018	163	5	4740	1	47	10	11
12.5	5655	533	3472	182214	2959	10789	165	175	38479	157	15	5117	11	42	14	9
13.5	4573	605	3045	49309	2434	9092	175	205	49389	145	2	4527	4	40	9	13
14.5	4232	451	3384	<1	1193	15916	181	176	105688	166	15	5062	14	58	14	9
15.5	5753	272	2883	<1	1250	18105	178	156	152031	169	40	4706	31	48	13	6
16.5	6443	535	2667	<1	1400	18566	184	140	136977	158	42	5650	34	55	10	6
17.5	4754	536	2867	<1	1550	15977	175	174	142708	163	36	5588	32	46	13	7
18.5	4429	473	2191	<1	1682	13462	166	157	87218	149	7	5468	1	55	15	12
19.5	5871	746	2362	<1	1933	15134	164	178	83055	177	50	6934	55	49	14	5
20.5	7303	481	2631	95133	2069	16785	162	170	80730	167	33	5903	10	62	14	9
21.5	6338	566	3227	<1	408	18366	185	144	121159	175	35	6473	37	53	19	6
22.5	6248	429	2720	363538	3472	11200	135	137	11140	154	42	6488	20	55	20	9
23.5	6752	432	2590	<1	2850	18053	194	165	138194	160	39	5772	24	55	17	7
24.5	5229	372	2626	<1	2133	13374	174	113	79409	159	8	4872	1	56	13	13
25.5	5948	499	3157	<1	107	16483	183	155	141303	158	5	5255	1	58	10	13
26.5	12639	616	2926	<1	420	17170	166	139	125519	172	43	5671	27	49	14	8
27.5	10989	244	2506	<1	1333	16141	166	157	111515	159	44	5392	16	54	18	7
28.5	8195	231	3138	<1	867	15830	165	185	120346	176	7	5299	1	55	12	13
29.5	17028	527	2313	124226	2733	15774	171	166	47883	168	18	5646	1	57	19	11
30.5	25171	510	3051	50329	1464	20843	172	144	95934	179	49	6011	19	65	18	6
31.5	17738	455	2506	338822	3496	13412	143	176	9096	176	34	6117	13	54	10	9
32.5	9241	629	2559	372782	2363	10937	125	126	5250	180	37	7600	15	56	10	9
33.5	7318	235	3235	134792	2930	13557	170	199	28664	150	1	4453	1	53	15	16
34.5	27987	812	3754	177584	1390	15428	149	106	100623	209	69	10924	45	57	10	4
35.5	3834	265	2691	389192	2178	8062	129	151	2262	154	11	4427	28	47	12	9
36.5	7339	705	6069	69959	1790	16275	169	128	87756	162	35	5862	48	53	14	5
37.5	4531	635	3998	138849	2806	14982	162	170	53110	150	14	4810	18	49	19	9
38.5	4849	674	4652	7324	1519	16674	157	169	106195	158	24	5098	40	51	8	7
39.5	9577	1369	19211	347977	3643	15392	147	105	17782	177	72	8875	103	52	12	2
40.5	7567	1138	14902	301539	3347	13468	146	136	25958	187	41	8060	55	53	11	3
41.5	6584	752	12686	266794	3266	13165	157	108	34824	176	33	6986	53	52	7	3
42.5	7271	1316	17715	362836	3638	12173	133	123	16945	151	38	9291	88	55	5	4
43.5	7813	1253	21756	283151	3047	14580	144	156	41087	179	33	9316	79	57	16	3
44.5	5518	729	14742	311516	3377	8726	155	171	23052	160	22	5759	32	46	13	6
45.5	6122	758	14326	221723	3043	10432	155	152	40229	161	28	5978	39	45	9	5
46.5	5086	862	11354	265982	3042	13499	143	145	41760	174	34	7074	61	48	11	6
47.5	5085	489	12155	234530	2994	8178	148	129	38433	157	28	5746	39	40	11	5
48.5	3561	298	8664	<1	1122	6713	178	154	118218	170	13	4003	14	32	13	7
49.5	12188	1327	38095	377758	3842	8568	130	104	24630	181	38	10932	58	53	3	3
50.5	9321	774	36499	380439	3673	6582	<1	111	4404	304	20	11043	16	59	2	6
51.5	6740	539	18136	256495	74	3326	<1	105	31631	270	23	9050	23	48	20	5
52.5	5595	705	20388	57413	1697	8866	<1	127	101986	391	19	4863	21	34	9	6
53.5	14506	943	40991	285401	2716	4913	<1	89	12835	287	29	8431	24	57	4	7
54.5	9207	573	19289	241717	308	3530	<1	138	37616	294	15	5510	2	49	4	12
56.5	17212	967	46195	306063	3563	5287	<1	105	7541	316	47	7300	29	58	2	5
58.0	17860	1049	47266	215849	2003	4927	76	99	27109	293	45	6172	29	49	93	3
60.0	17602	1012	45363	305174	2225	4104	109	119	11571	312	51	4396	23	65	108	7
71.0	26913	561	48159	67762	1274	6500	84	136	46345	276	98	2101	3	37	107	3
81.0	17383	2639	29009	213122	3349	10299	92	88	11192	258	120	19880	75	48	78	1
91.0	12749	2392	27956	145760	2415	7757	98	93	31944	235	101	21267	84	36	78	1
100.0	16301	6477	5781	317682	error	5407	<1	98	36312	182	63	10276	61	54	12	1
105.0	27166	6510	4775	289531	error	5896	<1	85	31268	178	79	10987	77	37	11	2
110.0	14534	6255	12477	260814	error	2280	<1	72	50620	177	56	11777	47	32	16	2
115.0	11737	6356	34253	158905	error	4041	<1	96	43012	130	78	21313	164	54	12	1
120.0	14771	5733	40809	171053	error	2567	<1	96	51991	139	74	21671	79	46	2	1
130.0	12843	5960	38843	110489	error	3103	<1	101	52599	124	87	26369	92	54	3	2
151.0	8769	2077	112811	205257	4863	8507	94	83	3888	144	73	49555	72	54	16	1
161.0	9794	2243	72503	217994	4563	10570	82	105	4277	189	91	39595	127	55	22	2
171.0	10631	1830	59990	242553	3575	8608	58	106	8166	222	83	26298	144	43	27	1
181.0	12572	2508	60777	232300	4529	11392	67	90	4918	235	74	35033	103	51	18	2
191.0	13522	2112	61870	231984	4139	10255	68	88	10317	258	74	32731	98	58	13	3
234.0	16801	417	2368	313358	3359	10680	<1	117	15607	376	29	4874	22	47	6	11
236.0	9820	854	18441	312881	3389	17569	121	105	45578	392	77	6663	97	51	115	4
239.0	20909	1177	11996	305734	3530	14865	128	126	19116	333	73	2966	104	46	101	4
241.0	12427	1046	16600	308338	3665	13350	103	128	49325	351	59	4847	117	47	128	4

depth	Ca	Ti	S	Si	Na	K	Cr	Zn	Mg	Mn	Sr	Fe	Zr	Rb	Ni	As
[cm]	[mg kg-1]															
243.0	7704	808	11962	319766	3476	11945	122	113	27401	319	61	4130	101	32	108	6
245.5	5108	389	5641	<1	3460	11176	<1	143	156471	401	11	4184	19	42	10	10
247.0	7038	1781	18963	335503	3445	15196	99	59	8541	332	57	7178	183	40	105	2
248.5	6552	2790	17482	263128	3328	17971	135	110	72263	358	66	4554	296	46	132	3
250.0	5363	1931	16087	268960	3152	16277	<1	60	44291	330	80	7840	256	51	14	1
251.0	7084	2263	21749	289886	3188	16251	<1	84	62926	363	62	8993	252	57	24	4
251.5	7511	1957	29947	364300	3801	14175	103	99	22233	338	54	10406	176	46	90	2
253.0	3850	1057	13025	389076	2929	8699	0.9	120	2206	310	53	5188	133	42	5	5
254.0	5184	1740	14795	227872	2941	14573	147	102	39349	389	37	<1	182	38	144	5
256.5	5381	1244	19492	270036	3311	12331	154	112	28997	357	40	<1	119	34	123	3
257.5	12109	862	45965	324028	2893	6277	59	99	9497	370	57	12222	86	33	58	2
260.0	9373	1728	31882	359035	3694	12430	<1	110	20365	362	44	9082	129	37	16	3
261.0	8258	1819	33109	369701	3795	14374	94	88	15940	343	63	10568	163	48	96	2
263.0	5854	1458	17815	265068	3237	15444	<1	111	60526	353	56	6819	127	48	11	2
265.0	8497	1706	33118	370197	3938	13571	99	75	22339	321	74	11285	182	43	81	1
267.0	5786	733	25537	363200	3586	7880	114	117	17855	305	52	4223	84	38	82	2
269.0	4215	436	6149	<1	809	13315	3	168	131364	403	26	4094	24	41	18	8
271.0	6101	766	19950	350224	3478	10805	108	123	13956	312	64	2395	78	23	102	1
272.5	6659	1523	28501	360305	3701	13483	104	125	20496	337	66	7751	202	52	93	3
275.0	5701	621	8287	59627	2225	14938	0.9	111	57476	384	39	4535	45	53	14	6
277.0	10242	1654	35144	371572	3955	12182	111	101	9913	363	74	13749	175	51	69	2
279.0	8335	1654	34672	367173	3853	13772	94	102	30574	358	69	10845	200	43	87	1
281.0	7614	1542	40790	360757	3696	13048	110	109	14589	340	67	10624	178	44	95	1
283.0	11471	2253	81442	340495	4489	11772	57	65	24446	522	45	31298	149	70	44	3
285.0	10036	1994	80023	293935	4388	10625	51	109	6399	546	43	37031	138	78	36	2
287.0	8473	1975	92777	356058	4179	11263	66	77	15232	523	36	32151	152	71	46	3
289.0	6230	1776	90801	376976	4004	9239	79	70	19359	533	32	36232	127	60	61	3
291.0	6973	1523	97972	353530	3885	5922	46	58	29827	748	40	55067	75	60	21	1
293.0	7524	1508	93647	365552	3735	5615	56	59	26988	765	44	57781	66	61	31	2
297.0	7564	1453	96766	331319	4302	5957	36	61	14780	854	20	55311	49	75	15	3
299.0	7634	1521	96101	334538	4387	6573	31	61	11322	804	27	54499	64	85	9	3
320.0	9732	6315	46525	220035	error	3231	<1	90	35482	206	46	22524	138	65	6	1
340.0	13949	6457	45174	196305	error	3598	<1	91	34001	246	45	30272	115	73	<1	1
360.0	11606	1414	101086	164618	4387	4651	66	74	5183	130	96	48220	81	44	<1	4
371.0	12848	2318	51846	271372	3993	11221	70	95	6087	246	118	25432	210	61	36	3
381.0	13212	2204	68404	226715	4676	10722	67	95	4703	220	108	37122	133	77	13	4
391.0	13537	2389	59477	232314	4224	10406	67	89	5593	248	92	33155	152	70	22	2
399.0	10144	1971	47524	321894	4157	11215	67	90	7815	249	79	22725	247	56	29	3
beach	9760	387	3823	144954	2878	8690	14	168	43197	376	50	3617	37	50	7	3
beach	11211	313	3124	<1	843	8991	<1	133	126749	399	35	3620	34	31	16	4
beach	11780	449	4172	<1	721	9808	<1	145	145065	444	41	4752	40	31	19	5
dune	9846	831	3812	<1	131	6821	<1	98	145127	460	27	4406	30	28	10	3
dune	59368	558	3148	184982	2411	9896	<1	88	76966	373	66	6352	37	48	9	4
dune	9827	237	4242	91990	2684	5130	25	139	47167	357	52	4127	51	37	7	3

Supplementary Table 4: Description of sediment types identified in thin section analysis. FOV – field of view; OM – organic matter. Subdivision of type 3 sediments can be achieved only in microscale.

Unit	Microstructure (physical appearance: size, shape, arrangement of primary particles, their clusters or compound particles and voids)	Scan of representative part of the unit	Matrix	Skeletal grains, size, shape, grading	Voids	Organic matter (OM)	Lower contact
type 3	chitonic microstructure: dense matrix of fine sand embedding coarse size skeleton grains, additionally clusters of clay and decomposed OM (clay-OM),<br>skeletal grains and clay-OM clusters are distributed and oriented along parallel lines, creating dipping laminae,<br>skeletal grains are coated and bridged by clay-OM, additionally clay-OM creates elongated, horizontal clusters	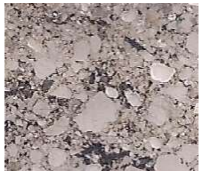	very fine densely packed sand with decomposed OM and clay of cloudy aspect	compound grains,<br>medium to coarse,<br>no grading,<br>subrounded to rounded,	compound,<br>interconnected packing voids,<br>c. 10% of FOV	OM if present, is completely decomposed,<br>occurs as cloudy aspect of voids or rarely in clusters within voids,<br>c. 5% of FOV	sharp, erosional, undulating, marked by the presence of matrix, associated with presence of elongated clusters of OM mixed with clay
	massive, apedal, clast supported microstructure, unimodal sized grains, no fine material in intergranular spaces, grains seem loose, touching each other	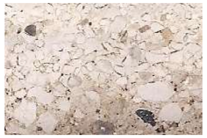	no material in interstitial pores				
type 2	very coarse to medium and fine size sand grains embedded within silt and fine sand matrix with clay-OM;<br>there are two types of texture:<br>i) skeletal grains of medium to coarse size are embedded within dense matrix;<br>ii) clusters of clay and decomposed OM filling voids, separated from the walls of voids, textural pedofeatures	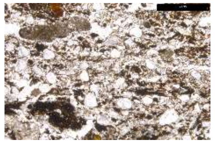	silt and fine sand with admixture of clay-OM	very coarse size to granule,<br>well sorted,<br>no grading,<br>subrounded to rounded,<br>c. 60-80 % of FOV	compound packing voids, vughs and occasional channels,<br>mainly horizontal,<br>c. 15% of FOV	decomposed<br>i) of very dark brown to black color, creates clusters within voids and<br>ii) having cloudy appearance fills interstitial pores between matrix grains,<br>c. 15% of FOV	sharp but not erosional, undulating, marked by the presence of matrix
type 1	silt to fine sand embedded within matrix consisting mixed clay and decomposed OM;<br>there are two types of texture:<br>i) clast supported fabric comprising grains of silt and fine sand with decomposed OM within interstitial voids;<br>ii) displaced coatings composed of decomposed OM and clay of crescentic appearance, textural pedofeatures	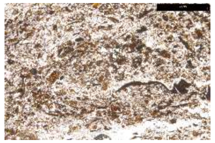	mixed clay and decomposed OM	silt to fine sand,<br>matrix supported,<br>well sorted,<br>subrounded to rounded	compound packing voids,<br>sinusoidal and crescentic channels	mixed with clay creates displaced coatings of crescentic shape,<br>oriented (sub)horizontal,<br>distributed roughly in laminae,<br>in places creates labyrinth microstructure	gradual,<br>marked with the presence of displaced clay coatings

Supplementary Table 5: Diatom count results, core M12 from Mechelinki.

taxon/depth [cm]	16	106	155	185	238	255	274.5	276	279
Amphora pediculus				43	84	57	48	86	78
Chamaepinnularia krookiformis			36	1					
Cocconeis disculus		1	1	60	39	45	45	41	77
Hippodonta hungarica				3	20	12		21	
Karayevia clevei				32	51	43	16	33	43
Mayamaea atomus var. permitis				3	21	8	3	6	3
Planothidium frequentissimum				28	69	60	46	35	27
Planothidium joursacense				52	72	68	38	146	30
Planothidium rostratum				56	42	85	55	45	77
Psammothidium lauenburgianum				3	6	4	10	4	5
Psammothidium subatomoides				2	19	16	7	12	14
Pseudostaurosira brevistriata			2	27	10	15	29	2	20
Staurosira construens	1		2	27	3	10	28	1	12
Staurosirella leptostauron			2	45	6	12		5	39
Staurosirella martyi		1	1	60	19	31	60	6	41

Supplementary Table 6: Heavy mineral counts, core M12, Mechelinki

depth [cm]	105	180	250	275	dune sample	beach sample
% content of the total fraction 125-250 ɥm	0.4	0.5	0.3	0.3	8.2	0.8
Tourmaline	1.0	4.8	4.2	1.1	0.9	0.4
Garnet	13.1	15.2	11.6	22.8	39.9	18.0
Amphibole	26.6	24.2	34.5	27.8	10.1	18.4
Epidote	13.1	13.9	11.8	9.2	4.4	10.2
Clinozoisite + Zoisite	1.0	2.4	2.2	1.1	0.6	0.4
Chlorite	0.5	0.6	2.7	0.6	0.3	4.3
Orthopyroxene	5.5	2.8	3.0	3.1	0.9	2.0
Cilnopyroxene	7.0	2.8	3.4	2.5	0.9	2.4
Staurolite	2.0	1.8	0.7	0.6	0.3	1.6
Carbonate minerals	0.0	0.6	1.5	0.3	0.9	2.0
Sillimanite + Andalusite	1.5	1.0	1.5	1.1	1.5	1.6
Kyanite	4.0	1.4	0.5	1.7	0.3	1.2
Zircon	2.0	2.2	0.5	3.1	1.2	1.2
Biotite+ Muscovite	1.0	0.4	4.7	0.3	0.0	5.5
Rutile	1.0	2.0	0.2	0.6	1.2	0.4
Titanite	1.0	2.8	1.5	1.4	0.6	0.4
Limonite	5.5	5.1	7.9	7.8	2.1	11.4
Opaque minerals	13.6	16.0	7.6	14.4	33.1	18.0
sum transparent minerals	80.9	78.8	84.5	77.8	64.8	70.6

Supplementary Table 7: Radiocarbon dating results. Asterisks indicate samples included in the age-depth model.

Lab No	Sediment depth [cm] *Compensated for mechanical and natural compaction*	Material	Raw AMS ^{14}C [years BP]	2σ calibrated years ranges [years BP]
Beta - 559088	62–63* *60-61*	bulk organic material	210±30	1–26; 142–221; 261–307
Poz-130620	98–100 *91-93*	bulk organic material	2550±30	2154–2263; 2296–2340
Poz-130619	112–114 *104-106*	bulk organic material	3120±30	3241–3399; 3431–3441
Poz-132355	150–151 *137-138*	bulk organic material	1540±30	1352–1517
Poz-130622	155–157* *142-144*	wood	640±30	554–665
Poz-132354	160–161 *147-148*	bulk organic material	3060±35	3172–3362
Poz-132356	185–186* *178-179*	bulk organic material	2905±30	2958–3158
Beta - 559089	188–189* *181-182*	bulk organic material	2910±30	2961–3159
Beta - 559090	238–239* *228-229*	bulk organic material	3430±30	3575–3726; 3747–3769; 3793–3823
Poz-130623	275–277* *264-266*	bulk organic material	3325±30	3460–3635
Poz-130624	288–290* *278-280*	bulk organic material	3345±30	3482–3639; 3667–3684
Beta - 559091	298–299* *288-289*	bulk organic material	4740±30	5328–5384; 5447–5581
GdA-6389	314–315* *300-301*	bulk organic material	5060±40	5663–5677; 5714–5911
GdA-6390	380–381* *360-361*	bulk organic material	6330±40	7163–7325; 7403–7409
GdA-6391	405–407* *395-397*	bulk organic material	6650±40	7431–7582

Supplementary Table 8: Table with depth and ages of sand layers.

upper boundary of sandy layer [cm]	lower boundary of sandy layer [cm]	minimum age	maximum age	median maximum age	median maximum age
17	21	-37	91	11	91
41	42	42	211	109	211
43	44	46	214	113	214
45	46	50	218	117	218
47	48	53	223	121	223
57	58	113	273	163	273
100	110	298	486	398	486
154	155	566	659	628	659
157	158	613	715	644	715
162	164	700	1048	872	1048
181	185	2856	3058	2974	3058
196	198	3044	3302	3160	3302
212	215	3231	3497	3367	3497
216	218	3247	3515	3384	3515
223	225	3324	3561	3457	3561
228	232	3399	3583	3498	3583
236	238	3486	3608	3564	3608
243	245	3506	3635	3580	3635
247	250	3512	3640	3585	3640
253	256	3520	3653	3594	3653
259	261	3528	3661	3602	3661
262.5	267	3533	3667	3607	3667
272	275	3552	3678	3625	3678
289	298	5162	5378	5203	5378

Supplementary Table 9: Table with coordinates of end member samples from modern sedimentary environments.

ID of the sample	location	geographical coordinates	analyses undertaken
SZ1	swash zone	54.62781N 18.51184E	grain size<br>geochemistry
SZ2	swash zone	54.62779N 18.51184E	grain size
LB1	lower beach	54.62784N 18.51170E	grain size<br>geochemistry<br>heavy minerals
LB2	lower beach	54.67782N 18.51168E	grain size
UB1	upper beach	54.62786N 18.51153E	grain size<br>geochemistry
D1	beach ridge and initial dune	54.62791N 18.51136E	grain size<br>geochemistry
D2	beach ridge and initial dune	54.62782N 18.51126E	grain size<br>geochemistry<br>heavy minerals
D3	beach ridge and initial dune	54.62783N 18.51122E	grain size<br>geochemistry

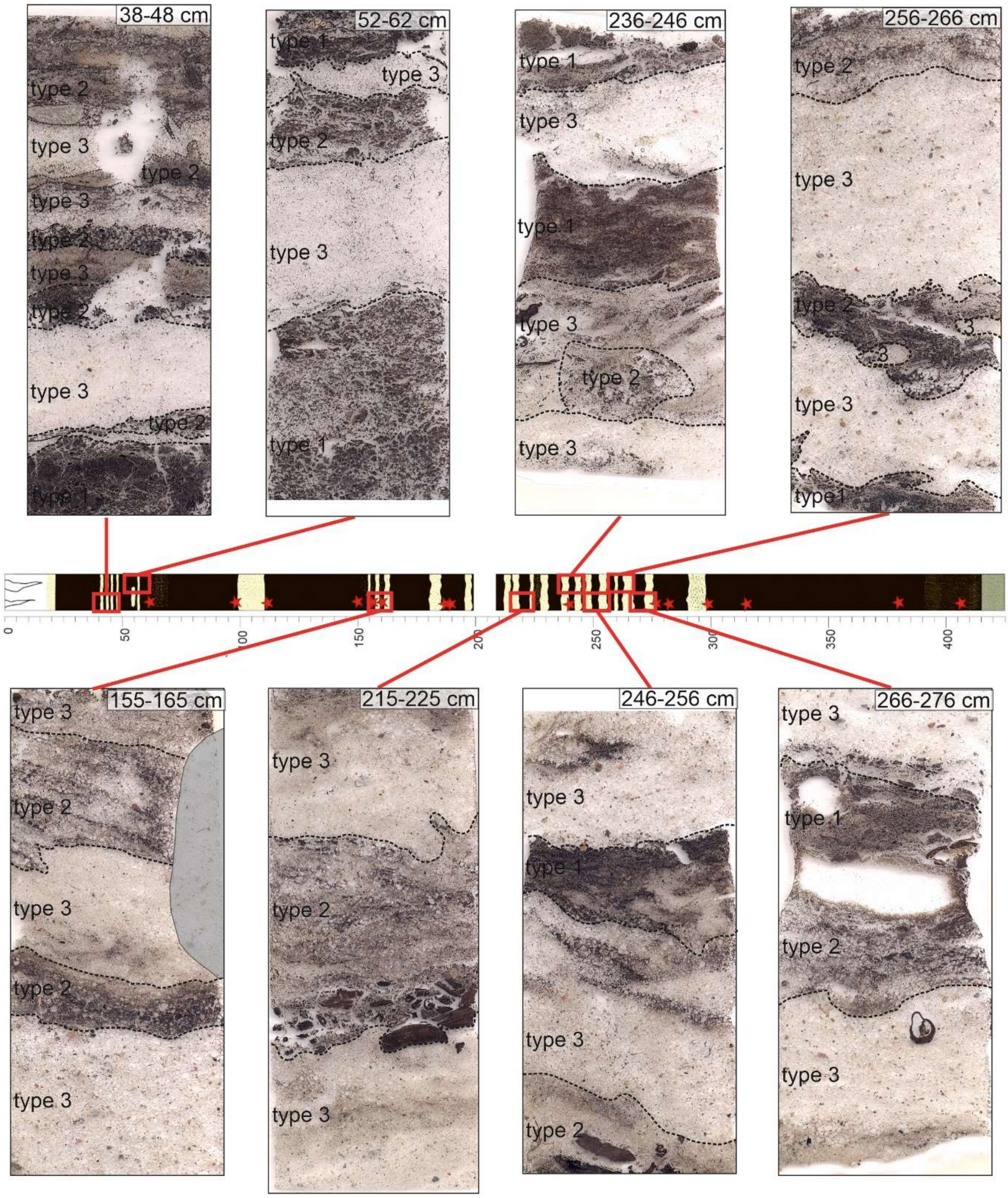

Supplementary Fig. 1: Micromorphological analysis results. Scans of thin sections of the selected fragments of master core M12. Blue rectangles indicate lower boundaries of event layers with scouring features and rip-up clasts. Detailed description of micromorphological units type 1 - 3 are presented in the Supplementary Table 4. The key to symbols of the core log is presented in Fig. 1 and 3.

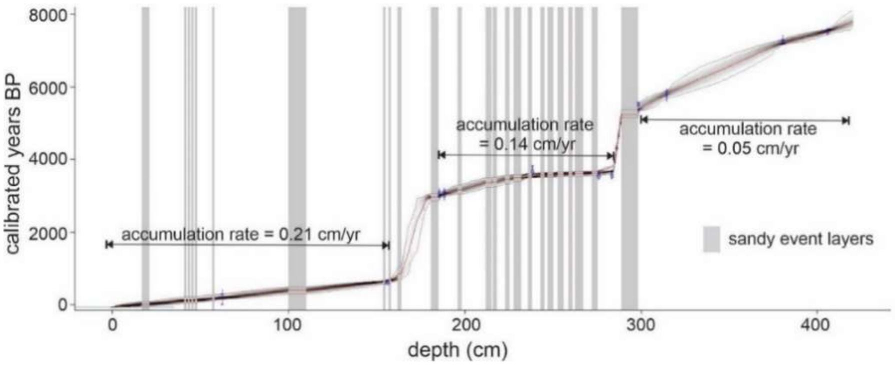

Supplementary Fig. 2: Age-depth model of master core M12 from Mechelinki. The sandy event layers are considered to be instantaneous deposits and are marked in gray.
